# *cyclo*-Tetra­kis{μ-*N*′-[(8-oxidoquinolin-7-yl)methyl­idene]isonicotino­hydrazidato}tetra­zinc tetra­hydrate

**DOI:** 10.1107/S1600536812018995

**Published:** 2012-05-05

**Authors:** Xiang-Wen Wu, Qing-Long Li, Jian-Ping Ma, Yu-Bin Dong

**Affiliations:** a College of Chemistry, Chemical Engineering and Materials Science, Key Laboratory of Molecular and Nano Probes, Engineering Research Center of Pesticide and Medicine Intermediate Clean Production, Ministry of Education, Shandong Provincial Key Laboratory of Clean Production of Fine Chemicals, Shandong Normal University, Jinan 250014, People’s Republic of China

## Abstract

In the title compound, [Zn_4_(C_16_H_10_N_4_O_2_)_4_]·4H_2_O, the *N*′-[(8-oxidoquinolin-7-yl)methyl­idene]isonicotinohydrazidate (*L* ^2−^) ligand binds to the metal ions, forming stable five- and six-membered chelate rings, leaving the pyridyl groups free. The compound is a tetra­nuclear Zn^II^ complex centered about a fourfold roto-inversion axis, with the ligand coordinating in the doubly deprotonated form. The Zn^II^ atom has a distorted square-pyramidal geometry being coordinated by one N and two O-atom donors from the doubly deprotonated *L* ^2−^ ligand, and by one N atom and one O-atom donor from a symmetry-related *L* ^2−^ ligand. In the crystal, four symmetry-related lattice water mol­ecules, centred about a fourfold roto-inversion axis, form a cyclic tetra­mer through O—H⋯O hydrogen bonds. These tetra­mers connect to the complex mol­ecules through O—H⋯N hydrogen bonds, forming a chain propagating along [100]. Neighbouring mol­ecules are linked by π–π inter­actions [centroid–centroid distance = 3.660 (2) Å] involving the quinolidine rings.

## Related literature

For heterometallic coordination polymers and coordination compounds involving bridging *N*-donor ligands, see: Palacios *et al.* (2008 ▶); Tao *et al.* (2002 ▶); Dong *et al.* (2005 ▶). For details of bond lengths in similar zinc(II) complexes, see: Kumar *et al.* (2006 ▶); Woodward *et al.* (2006 ▶).
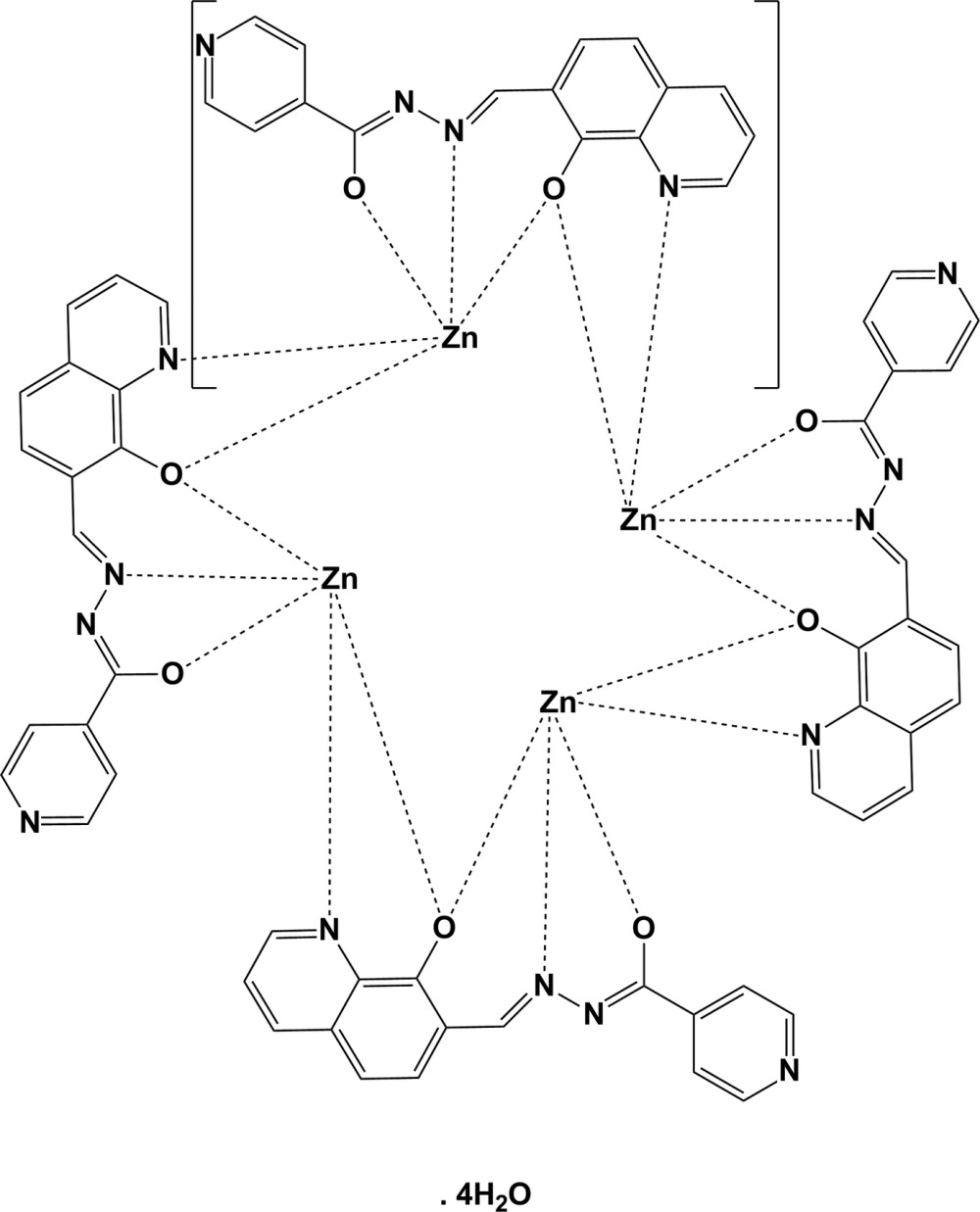


## Experimental

### 

#### Crystal data

[Zn_4_(C_16_H_10_N_4_O_2_)_4_]·4H_2_O
*M* *_r_* = 1494.66
Tetragonal, 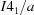

*a* = 21.407 (2) Å
*c* = 13.626 (3) Å
*V* = 6244.1 (15) Å^3^
*Z* = 4
Mo *K*α radiation
μ = 1.60 mm^−1^
*T* = 298 K
0.13 × 0.11 × 0.07 mm

#### Data collection

Bruker SMART CCD area-detector diffractometer
Absorption correction: multi-scan (*SADABS*; Bruker, 2003 ▶) *T*_min_ = 0.819, *T*_max_ = 0.897
16035 measured reflections
2900 independent reflections
2324 reflections with *I* > 2σ(*I*)
*R*_int_ = 0.054

#### Refinement

*R*[*F* ^2^ > 2σ(*F* ^2^)] = 0.036
*wR*(*F* ^2^) = 0.087
*S* = 1.03
2900 reflections
217 parameters
H-atom parameters constrained
Δρ_max_ = 0.27 e Å^−3^
Δρ_min_ = −0.23 e Å^−3^

### 

Data collection: *SMART* (Bruker, 2003 ▶); cell refinement: *SAINT* (Bruker, 2003 ▶); data reduction: *SAINT*; program(s) used to solve structure: *SHELXS97* (Sheldrick, 2008 ▶); program(s) used to refine structure: *SHELXL97* (Sheldrick, 2008 ▶); molecular graphics: *SHELXTL* (Sheldrick, 2008 ▶); software used to prepare material for publication: *SHELXTL*.

## Supplementary Material

Crystal structure: contains datablock(s) I, global. DOI: 10.1107/S1600536812018995/su2415sup1.cif

Structure factors: contains datablock(s) I. DOI: 10.1107/S1600536812018995/su2415Isup2.hkl

Additional supplementary materials:  crystallographic information; 3D view; checkCIF report

## Figures and Tables

**Table 1:** Hydrogen-bond geometry (Å, °)

*D*—H⋯*A*	*D*—H	H⋯*A*	*D*⋯*A*	*D*—H⋯*A*
O3—H3*B*⋯N3	0.85	2.08	2.912 (4)	168
O3—H3*A*⋯O3^i^	0.85	1.99	2.835 (5)	173

Symmetry code: (i) 
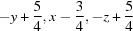
.

**Fig. 1.**
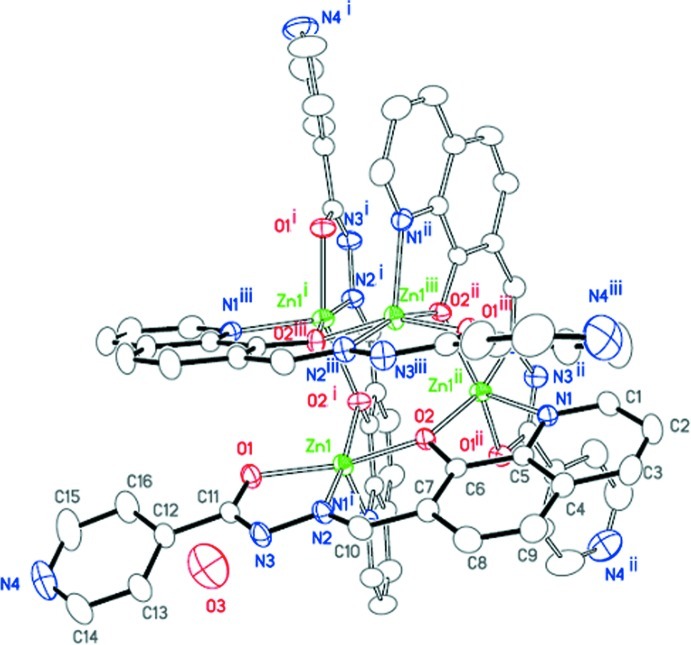
The molecular structure of the title ZnII complex. Displacement ellipsoids are drawn at the 30% probability level. H atoms have been omitted for clarity; only one of the symmetry related water molecules are shown; symmetry codes: (i) -y + 5/4, x - 3/4, -z + 9/4; (ii) y + 3/4, -x + 5/4, -z + 9/4; (iii) -x + 2, -y + 1/2, z.

**Fig. 2.**
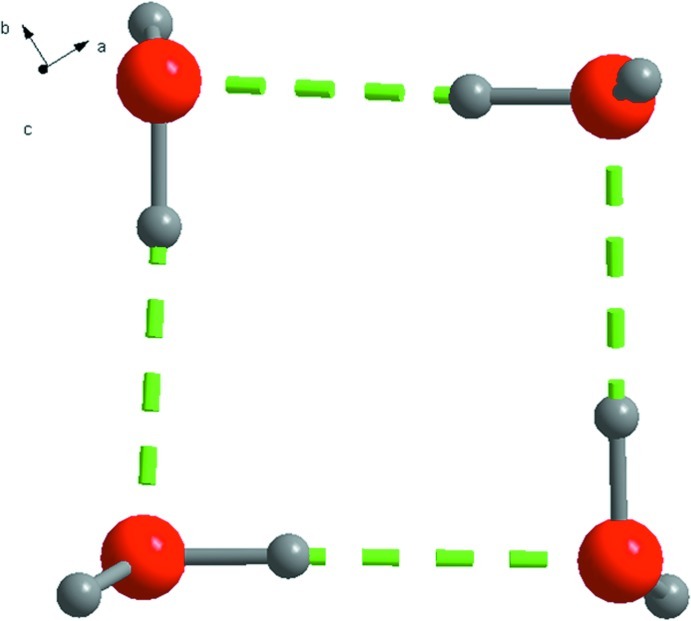
A view of the cyclic tetramer cluster formed between uncoordinated water molecules related by a four-fold roto-inversion axis.

**Fig. 3.**
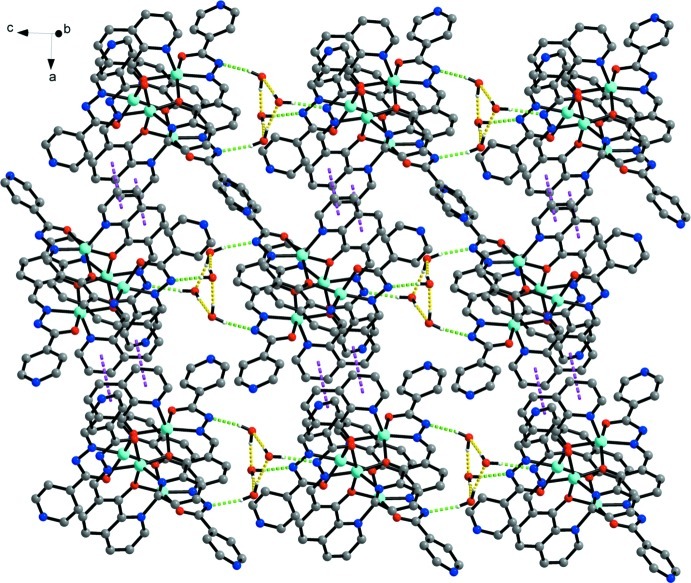
The two-dimensional supramolecular sheet of the title ZnII complex, formed by hydrogen bonds and π-π interactions (dashed lines) between two symmetry-related quinoline rings [centroid-to-centroid distance 3.660 (2) Å [symmetry code: -x+2, -y+1, -z+2].

## supplementary crystallographic information

### Comment

The synthesis of metal-containing compounds is the first and an important step in a promising route to novel heterometallic coordination polymers (Tao *et al.*, 2002). It is well known that the relative orientations of N donors and the variation of the bridging spacer may lead to the construction of supramolecular motifs that have not been achieved using normal linear organic ligands. The ligand N'-((8-hydroxyquinolin-7-yl)methylene)isonicotinohydrazide ligand (LH_2_) is unsymmetrical, containing two different terminal coordinating sites, *i.e.* a pyridyl and a 7-hydrazinylidene-8-hydroxyquinoline chelator. The latter contains the N/O-bidentate chelating motif, which usually binds to metal ions in a deprotonated manner (Palacios *et al.*, 2008). It was also found that this chelator binds to metal ions in preference to the pyridine N atom. This could provide a favourable coordination strategy for the synthesis of multinuclear metal-containing compounds. As part of our continuing studies of coordination compounds with bridging N-donor ligands (Dong *et al.*, 2005), we report herein on the synthesis and crystal structure of a novel Zn^II ^compound with free pyridyl groups.

The title compound is a tetranuclear Zn^II^ complex, centred about a fourfold roto-inversion axis, and crystallizes as a tetrahydrate (Fig. 1). The Zn^II^ atom has distorted square-pyramidal geometry, being coordinated by one N (N2) and two O donors (O1 and O2) from a doubly deprotonated LH_2_ ligand, and one N (N1^i^) and one O donor (O2^i^) from a symmetry-related *L*^2-^ ligand [symmetry code :(i) -*y* + 5/4, *x* - 3/4, -*z* + 9/4]. The N atoms of the pyridine rings are not involved in coordination. The dihedral angle between the pyridine and quinoline ring mean planes is 14.01 (15)^°^. The Zn—N distances are 2.081 (2) for N1^i^ and 2.036 (2) Å for N2, which are consistent with values reported previously (Kumar *et al.*, 2006). The Zn—O bond lengths, 2.0329 (19) Å for O1, 2.0350 (18) Å for O2^i ^and 2.0604 (18) Å for O2, are very close to the Zn—O bond lengths reported by (Woodward *et al.*, 2006).

In the crystal, four symmetry-related lattice water molecules form a cyclic tetramer through O—H···O hydrogen bonds (Fig. 2 and Table 1). These water tetramers are linked to the complex molecules through O—H···N hydrogen bonds (Table 1), so forming a one-dimensional chain propagating parallel to the [001] direction. Parallel chains are connected by π-π interactions involving rings (C4—C9) and (N1/C1—C5)^ii ^[centroid-to-centroid distance 3.660 (2) Å; symmetry code: (iv) -*x+2*, -*y+1*, -*z+2*] resulting in the formation of a two-dimensional network (Fig. 3).

### Experimental

A solution of LH_2_ (5.3 mg,0.02 mmol) in MeOH (8 ml) was layered onto a solution of ZnSO_4_ (5.8 mg, 0.04 mmol) in water (8 mL). The system was left for about two weeks at room temperature and yellow crystals of the title complex were obtained (yield 5.6 mg, 79%). Analysis, calc. for C_64_H_48_N_16_O_12_Zn_4_: *C* 51.43, H 3.24, N 14.99%; found: C 51.39, H 3.30, N 14.93%.

### Refinement

The C-bound H atoms were placed in geometrically idealized positions and included as riding atoms: C—H = 0.93 Å and U_iso_(H) = 1.2U_eq_(C). The water H atoms were located in a difference Fourier maps and refined with distance O—H restrained to 0.85 (2) Å and U_iso_(H) = 1.2U_eq_(O)

### Crystal data

**Table:**

[Zn_4_(C_16_H_10_N_4_O_2_)_4_]·4H_2_O	*D*_x_ = 1.590 Mg m^−^^3^
*M**_r_* = 1494.66	Mo *K*α radiation, λ = 0.71073 Å
Tetragonal, *I*4_1_/*a*	Cell parameters from 2897 reflections
Hall symbol: I 41/a	θ = 2.6–22.7°
*a* = 21.407 (2) Å	µ = 1.60 mm^−^^1^
*c* = 13.626 (3) Å	*T* = 298 K
*V* = 6244.1 (15) Å^3^	Block, yellow
*Z* = 4	0.13 × 0.11 × 0.07 mm
*F*(000) = 3040	

### Data collection

**Table:**

Bruker SMART CCD area-detector diffractometer	2900 independent reflections
Radiation source: fine-focus sealed tube	2324 reflections with *I* > 2σ(*I*)
Graphite monochromator	*R*_int_ = 0.054
phi and ω scans	θ_max_ = 25.5°, θ_min_ = 1.8°
Absorption correction: multi-scan (*SADABS*; Bruker, 2003)	*h* = −22→25
*T*_min_ = 0.819, *T*_max_ = 0.897	*k* = −25→25
16035 measured reflections	*l* = −13→16

### Refinement

**Table:**

Refinement on *F*^2^	Primary atom site location: structure-invariant direct methods
Least-squares matrix: full	Secondary atom site location: difference Fourier map
*R*[*F*^2^ > 2σ(*F*^2^)] = 0.036	Hydrogen site location: inferred from neighbouring sites
*wR*(*F*^2^) = 0.087	H-atom parameters constrained
*S* = 1.03	*w* = 1/[σ^2^(*F*_o_^2^) + (0.0372*P*)^2^ + 5.0654*P*] where *P* = (*F*_o_^2^ + 2*F*_c_^2^)/3
2900 reflections	(Δ/σ)_max_ = 0.001
217 parameters	Δρ_max_ = 0.27 e Å^−^^3^
0 restraints	Δρ_min_ = −0.23 e Å^−^^3^

### Special details

**Table:**

Geometry. All e.s.d.'s (except the e.s.d. in the dihedral angle between two l.s. planes) are estimated using the full covariance matrix. The cell e.s.d.'s are taken into account individually in the estimation of e.s.d.'s in distances, angles and torsion angles; correlations between e.s.d.'s in cell parameters are only used when they are defined by crystal symmetry. An approximate (isotropic) treatment of cell e.s.d.'s is used for estimating e.s.d.'s involving l.s. planes.
Refinement. Refinement of *F*^2^ against ALL reflections. The weighted *R*-factor *wR* and goodness of fit *S* are based on *F*^2^, conventional *R*-factors *R* are based on *F*, with *F* set to zero for negative *F*^2^. The threshold expression of *F*^2^ > σ(*F*^2^) is used only for calculating *R*-factors(gt) *etc*. and is not relevant to the choice of reflections for refinement. *R*-factors based on *F*^2^ are statistically about twice as large as those based on *F*, and *R*- factors based on ALL data will be even larger.

### Fractional atomic coordinates and isotropic or equivalent isotropic displacement parameters (Å^2^)

**Table:**

	*x*	*y*	*z*	*U*_iso_*/*U*_eq_	
C1	1.10071 (14)	0.45462 (15)	1.1624 (3)	0.0485 (8)	
H1	1.1119	0.4530	1.2283	0.058*	
C2	1.12416 (16)	0.50312 (16)	1.1052 (3)	0.0611 (10)	
H2	1.1506	0.5329	1.1324	0.073*	
C3	1.10790 (16)	0.50630 (15)	1.0094 (3)	0.0590 (10)	
H3	1.1230	0.5388	0.9707	0.071*	
C4	1.06842 (14)	0.46097 (14)	0.9677 (3)	0.0442 (8)	
C5	1.04647 (12)	0.41352 (13)	1.0312 (2)	0.0336 (6)	
C6	1.00568 (12)	0.36577 (12)	0.9960 (2)	0.0303 (6)	
C7	0.98754 (13)	0.36698 (13)	0.8981 (2)	0.0361 (7)	
C8	1.01152 (15)	0.41496 (15)	0.8363 (2)	0.0497 (8)	
H8	1.0003	0.4149	0.7704	0.060*	
C9	1.04951 (15)	0.46024 (16)	0.8689 (3)	0.0538 (9)	
H9	1.0633	0.4911	0.8262	0.065*	
C10	0.94522 (13)	0.32259 (14)	0.8548 (2)	0.0394 (7)	
H10	0.9397	0.3241	0.7871	0.047*	
C11	0.84867 (13)	0.20093 (13)	0.9058 (2)	0.0374 (7)	
C12	0.80184 (14)	0.15938 (14)	0.8575 (3)	0.0453 (8)	
C13	0.77607 (18)	0.17239 (18)	0.7669 (3)	0.0684 (11)	
H13	0.7896	0.2065	0.7303	0.082*	
C14	0.7291 (2)	0.1330 (2)	0.7317 (4)	0.0865 (15)	
H14	0.7106	0.1434	0.6721	0.104*	
C15	0.7364 (2)	0.0697 (2)	0.8615 (4)	0.0953 (16)	
H15	0.7244	0.0332	0.8935	0.114*	
C16	0.78125 (17)	0.10645 (17)	0.9055 (3)	0.0641 (10)	
H16	0.7974	0.0958	0.9666	0.077*	
N1	1.06328 (10)	0.41084 (10)	1.12773 (18)	0.0352 (6)	
N2	0.91453 (11)	0.28117 (11)	0.90248 (18)	0.0356 (6)	
N3	0.87505 (12)	0.24302 (11)	0.84775 (18)	0.0418 (6)	
N4	0.70908 (18)	0.0828 (2)	0.7760 (3)	0.0948 (13)	
O1	0.85836 (9)	0.19375 (9)	0.99696 (16)	0.0407 (5)	
O2	0.98781 (8)	0.32250 (8)	1.06007 (13)	0.0321 (4)	
O3	0.90970 (15)	0.23053 (16)	0.6420 (2)	0.1038 (11)	
H3A	0.9434	0.2098	0.6371	0.125*	
H3B	0.8948	0.2309	0.6998	0.125*	
Zn1	0.910323 (15)	0.265779 (15)	1.04977 (2)	0.03319 (13)	

### Atomic displacement parameters (Å^2^)

**Table:**

	*U*^11^	*U*^22^	*U*^33^	*U*^12^	*U*^13^	*U*^23^
C1	0.0442 (18)	0.0485 (19)	0.053 (2)	0.0001 (15)	−0.0103 (16)	−0.0125 (16)
C2	0.055 (2)	0.048 (2)	0.080 (3)	−0.0168 (16)	−0.010 (2)	−0.009 (2)
C3	0.056 (2)	0.0398 (19)	0.081 (3)	−0.0139 (16)	0.004 (2)	0.0075 (19)
C4	0.0402 (17)	0.0385 (17)	0.054 (2)	−0.0054 (14)	0.0016 (15)	0.0072 (15)
C5	0.0317 (15)	0.0319 (15)	0.0371 (17)	0.0010 (11)	0.0006 (13)	0.0033 (13)
C6	0.0312 (14)	0.0295 (14)	0.0301 (16)	0.0012 (11)	0.0024 (12)	0.0056 (12)
C7	0.0407 (16)	0.0377 (16)	0.0299 (16)	−0.0028 (13)	0.0030 (13)	0.0026 (13)
C8	0.056 (2)	0.059 (2)	0.0336 (18)	−0.0082 (17)	−0.0018 (16)	0.0167 (16)
C9	0.058 (2)	0.052 (2)	0.051 (2)	−0.0124 (17)	0.0043 (17)	0.0212 (17)
C10	0.0448 (17)	0.0518 (18)	0.0214 (15)	−0.0025 (14)	−0.0032 (13)	0.0025 (14)
C11	0.0369 (16)	0.0375 (16)	0.0378 (18)	0.0022 (13)	−0.0078 (14)	−0.0077 (14)
C12	0.0392 (17)	0.0460 (18)	0.051 (2)	0.0003 (14)	−0.0057 (15)	−0.0151 (16)
C13	0.077 (3)	0.062 (2)	0.066 (3)	−0.008 (2)	−0.033 (2)	−0.008 (2)
C14	0.089 (3)	0.090 (3)	0.080 (3)	−0.004 (3)	−0.043 (3)	−0.022 (3)
C15	0.103 (4)	0.092 (3)	0.091 (4)	−0.053 (3)	−0.001 (3)	−0.016 (3)
C16	0.069 (2)	0.066 (2)	0.057 (3)	−0.0237 (19)	−0.004 (2)	−0.004 (2)
N1	0.0334 (13)	0.0337 (13)	0.0386 (15)	0.0000 (10)	−0.0030 (11)	−0.0050 (11)
N2	0.0406 (14)	0.0419 (14)	0.0244 (13)	−0.0053 (11)	−0.0040 (11)	−0.0006 (11)
N3	0.0492 (15)	0.0483 (15)	0.0279 (14)	−0.0088 (12)	−0.0102 (12)	−0.0038 (12)
N4	0.082 (3)	0.105 (3)	0.097 (3)	−0.035 (2)	−0.019 (2)	−0.029 (3)
O1	0.0476 (12)	0.0410 (11)	0.0335 (12)	−0.0098 (9)	−0.0072 (10)	−0.0006 (10)
O2	0.0395 (11)	0.0339 (10)	0.0228 (10)	−0.0075 (8)	−0.0038 (8)	0.0046 (8)
O3	0.128 (3)	0.144 (3)	0.0396 (17)	−0.007 (2)	0.0108 (17)	−0.0185 (19)
Zn1	0.0380 (2)	0.0382 (2)	0.02344 (19)	−0.00629 (14)	−0.00228 (14)	0.00205 (14)

### Geometric parameters (Å, º)

**Table:**

C1—N1	1.321 (4)	C11—C12	1.493 (4)
C1—C2	1.392 (5)	C12—C13	1.380 (5)
C1—H1	0.9300	C12—C16	1.381 (5)
C2—C3	1.353 (5)	C13—C14	1.397 (5)
C2—H2	0.9300	C13—H13	0.9300
C3—C4	1.406 (5)	C14—N4	1.305 (6)
C3—H3	0.9300	C14—H14	0.9300
C4—C9	1.406 (5)	C15—N4	1.333 (6)
C4—C5	1.414 (4)	C15—C16	1.379 (5)
C5—N1	1.365 (4)	C15—H15	0.9300
C5—C6	1.427 (4)	C16—H16	0.9300
C6—O2	1.329 (3)	N1—Zn1^i^	2.081 (2)
C6—C7	1.390 (4)	N2—N3	1.392 (3)
C7—C8	1.423 (4)	N2—Zn1	2.036 (2)
C7—C10	1.439 (4)	O1—Zn1	2.0329 (19)
C8—C9	1.341 (4)	O2—Zn1^i^	2.0350 (18)
C8—H8	0.9300	O2—Zn1	2.0605 (18)
C9—H9	0.9300	O3—H3A	0.8499
C10—N2	1.281 (3)	O3—H3B	0.8502
C10—H10	0.9300	Zn1—O2^ii^	2.0350 (18)
C11—O1	1.269 (4)	Zn1—N1^ii^	2.081 (2)
C11—N3	1.325 (4)		
			
N1—C1—C2	123.2 (3)	C12—C13—C14	118.2 (4)
N1—C1—H1	118.4	C12—C13—H13	120.9
C2—C1—H1	118.4	C14—C13—H13	120.9
C3—C2—C1	119.0 (3)	N4—C14—C13	125.1 (4)
C3—C2—H2	120.5	N4—C14—H14	117.5
C1—C2—H2	120.5	C13—C14—H14	117.5
C2—C3—C4	120.6 (3)	N4—C15—C16	124.4 (4)
C2—C3—H3	119.7	N4—C15—H15	117.8
C4—C3—H3	119.7	C16—C15—H15	117.8
C9—C4—C3	124.5 (3)	C15—C16—C12	119.0 (4)
C9—C4—C5	118.8 (3)	C15—C16—H16	120.5
C3—C4—C5	116.7 (3)	C12—C16—H16	120.5
N1—C5—C4	122.1 (3)	C1—N1—C5	118.4 (3)
N1—C5—C6	117.0 (2)	C1—N1—Zn1^i^	130.2 (2)
C4—C5—C6	120.8 (3)	C5—N1—Zn1^i^	111.07 (17)
O2—C6—C7	124.3 (2)	C10—N2—N3	116.5 (2)
O2—C6—C5	117.0 (2)	C10—N2—Zn1	129.4 (2)
C7—C6—C5	118.7 (2)	N3—N2—Zn1	113.97 (17)
C6—C7—C8	118.7 (3)	C11—N3—N2	109.7 (2)
C6—C7—C10	123.9 (3)	C14—N4—C15	115.7 (4)
C8—C7—C10	117.5 (3)	C11—O1—Zn1	110.12 (18)
C9—C8—C7	123.0 (3)	C6—O2—Zn1^i^	113.95 (16)
C9—C8—H8	118.5	C6—O2—Zn1	126.71 (17)
C7—C8—H8	118.5	Zn1^i^—O2—Zn1	114.03 (8)
C8—C9—C4	120.0 (3)	H3A—O3—H3B	113.4
C8—C9—H9	120.0	O1—Zn1—O2^ii^	102.00 (8)
C4—C9—H9	120.0	O1—Zn1—N2	78.35 (9)
N2—C10—C7	124.9 (3)	O2^ii^—Zn1—N2	164.76 (8)
N2—C10—H10	117.6	O1—Zn1—O2	155.73 (8)
C7—C10—H10	117.6	O2^ii^—Zn1—O2	87.94 (8)
O1—C11—N3	126.7 (3)	N2—Zn1—O2	86.34 (8)
O1—C11—C12	118.0 (3)	O1—Zn1—N1^ii^	97.94 (8)
N3—C11—C12	115.3 (3)	O2^ii^—Zn1—N1^ii^	80.24 (8)
C13—C12—C16	117.5 (3)	N2—Zn1—N1^ii^	114.92 (9)
C13—C12—C11	122.9 (3)	O2—Zn1—N1^ii^	105.61 (8)
C16—C12—C11	119.6 (3)		
			
N1—C1—C2—C3	0.4 (5)	C4—C5—N1—Zn1^i^	174.9 (2)
C1—C2—C3—C4	−0.7 (5)	C6—C5—N1—Zn1^i^	−5.5 (3)
C2—C3—C4—C9	−179.5 (3)	C7—C10—N2—N3	179.0 (3)
C2—C3—C4—C5	1.0 (5)	C7—C10—N2—Zn1	3.4 (4)
C9—C4—C5—N1	179.4 (3)	O1—C11—N3—N2	−2.2 (4)
C3—C4—C5—N1	−1.0 (4)	C12—C11—N3—N2	176.2 (2)
C9—C4—C5—C6	−0.2 (4)	C10—N2—N3—C11	177.3 (3)
C3—C4—C5—C6	179.4 (3)	Zn1—N2—N3—C11	−6.4 (3)
N1—C5—C6—O2	−0.4 (4)	C13—C14—N4—C15	0.4 (8)
C4—C5—C6—O2	179.2 (2)	C16—C15—N4—C14	2.6 (8)
N1—C5—C6—C7	180.0 (2)	N3—C11—O1—Zn1	9.4 (4)
C4—C5—C6—C7	−0.4 (4)	C12—C11—O1—Zn1	−169.0 (2)
O2—C6—C7—C8	−178.2 (3)	C7—C6—O2—Zn1^i^	−174.1 (2)
C5—C6—C7—C8	1.4 (4)	C5—C6—O2—Zn1^i^	6.3 (3)
O2—C6—C7—C10	2.3 (4)	C7—C6—O2—Zn1	−21.6 (4)
C5—C6—C7—C10	−178.1 (3)	C5—C6—O2—Zn1	158.79 (18)
C6—C7—C8—C9	−2.0 (5)	C11—O1—Zn1—O2^ii^	−173.51 (18)
C10—C7—C8—C9	177.6 (3)	C11—O1—Zn1—N2	−9.08 (19)
C7—C8—C9—C4	1.3 (5)	C11—O1—Zn1—O2	−61.1 (3)
C3—C4—C9—C8	−179.8 (3)	C11—O1—Zn1—N1^ii^	104.85 (19)
C5—C4—C9—C8	−0.2 (5)	C10—N2—Zn1—O1	−175.7 (3)
C6—C7—C10—N2	7.5 (5)	N3—N2—Zn1—O1	8.59 (18)
C8—C7—C10—N2	−172.0 (3)	C10—N2—Zn1—O2^ii^	−82.8 (4)
O1—C11—C12—C13	163.3 (3)	N3—N2—Zn1—O2^ii^	101.5 (3)
N3—C11—C12—C13	−15.3 (5)	C10—N2—Zn1—O2	−14.7 (3)
O1—C11—C12—C16	−14.5 (4)	N3—N2—Zn1—O2	169.66 (19)
N3—C11—C12—C16	166.9 (3)	C10—N2—Zn1—N1^ii^	90.9 (3)
C16—C12—C13—C14	2.8 (6)	N3—N2—Zn1—N1^ii^	−84.83 (19)
C11—C12—C13—C14	−175.1 (3)	C6—O2—Zn1—O1	73.9 (3)
C12—C13—C14—N4	−3.1 (7)	Zn1^i^—O2—Zn1—O1	−133.61 (16)
N4—C15—C16—C12	−2.7 (7)	C6—O2—Zn1—O2^ii^	−170.8 (2)
C13—C12—C16—C15	−0.2 (6)	Zn1^i^—O2—Zn1—O2^ii^	−18.38 (9)
C11—C12—C16—C15	177.8 (4)	C6—O2—Zn1—N2	23.3 (2)
C2—C1—N1—C5	−0.4 (4)	Zn1^i^—O2—Zn1—N2	175.75 (10)
C2—C1—N1—Zn1^i^	−173.3 (2)	C6—O2—Zn1—N1^ii^	−91.6 (2)
C4—C5—N1—C1	0.8 (4)	Zn1^i^—O2—Zn1—N1^ii^	60.89 (11)
C6—C5—N1—C1	−179.6 (2)		

Symmetry codes: (i) *y*+3/4, −*x*+5/4, −*z*+9/4; (ii) −*y*+5/4, *x*−3/4, −*z*+9/4.

### Hydrogen-bond geometry (Å, º)

**Table:**

*D*—H···*A*	*D*—H	H···*A*	*D*···*A*	*D*—H···*A*
O3—H3*B*···N3	0.85	2.08	2.912 (4)	168
O3—H3*A*···O3^iii^	0.85	1.99	2.835 (5)	173

Symmetry code: (iii) −*y*+5/4, *x*−3/4, −*z*+5/4.
